# Clinical, Biological and Genetic Analysis of Prepubertal Isolated Ovarian Cyst in 11 Girls

**DOI:** 10.1371/journal.pone.0011282

**Published:** 2010-06-25

**Authors:** Raja Brauner, Anu Bashamboo, Sébastien Rouget, Marie Goulet, Pascal Philibert, Hélène Sarda-Thibault, Christine Trivin, Micheline Misrahi, Charles Sultan, Ken McElreavey

**Affiliations:** 1 Université Paris Descartes and AP-HP, Hôpital Bicêtre, Unité d'endocrinologie pédiatrique, Le Kremlin Bicêtre, Paris, France; 2 Human Developmental Genetics, Institut Pasteur, Paris, France; 3 Hôpital Lapeyronie, Service d'hormonologie du développement et de la reproduction, Montpellier, France; 4 Centre hospitalier René Dubos, Service de Pédiatrie, Pontoise, France; 5 AP-HP, Hôpital Necker-Enfants Malades, Service d'explorations fonctionnelles, Paris, France; 6 Université Paris 11, INSERM U 854 and AP-HP, Hôpital Bicêtre, Laboratoire de génétique moléculaire, pharmacogénétique, hormonologie, Le Kremlin Bicêtre, Paris, France; Health Canada, Canada

## Abstract

**Background:**

The cause of isolated gonadotropin-independent precocious puberty (PP) with an ovarian cyst is unknown in the majority of cases. Here, we describe 11 new cases of peripheral PP and, based on phenotypes observed in mouse models, we tested the hypothesis that mutations in the *GNAS1*, *NR5A1*, *LHCGR*, *FSHR, NR5A1, StAR*, *DMRT4* and *NOBOX* may be associated with this phenotype.

**Methodology/Principal Findings:**

11 girls with gonadotropin-independent PP were included in this study. Three girls were seen for a history of prenatal ovarian cyst, 6 girls for breast development, and 2 girls for vaginal bleeding. With one exception, all girls were seen before 8 years of age. In 8 cases, an ovarian cyst was detected, and in one case, suspected. One other case has polycystic ovaries, and the remaining case was referred for vaginal bleeding. Four patients had a familial history of ovarian anomalies and/or infertility. Mutations in the coding sequences of the candidate genes GNAS1, NR5A1, LHCGR, FSHR, NR5A1, StAR, DMRT4 and NOBOX were not observed.

**Conclusions/Significance:**

Ovarian PP shows markedly different clinical features from central PP. Our data suggest that mutations in the GNAS1, NR5A1, LHCGR, FSHR StAR, DMRT4 and NOBOX genes are not responsible for ovarian PP. Further research, including the identification of familial cases, is needed to understand the etiology of ovarian PP.

## Introduction

Precocious puberty (PP) in girls is defined by the development of sexual characters (development of breasts, pubic and menstrual bleeding) and increased growth rate before 8 years-of-age [1]. There are two major categories of precocious puberty: true or central PP and pseudo or peripheral PP. True PP is associated with the premature activation of the hypothalamic-pituitary-gonadal axis [1], [2]. Peripheral PP is characterized by the development of secondary sexual characteristics mainly due to estradiol secretion originating from either adrenals or from ovaries. True and peripheral isosexual PP can be distinguished by measuring basal, and gonadotropin releasing hormone (GnRH)-stimulated luteinising hormone (LH) and follicle stimulating hormone (FSH) peaks concentrations. These concentrations increase in true PP [3] while they are low and do not increase in peripheral PP.

Peripheral PP of ovarian origin is a rare condition compared to true PP. It may be associated with an ovarian cyst and it is often transient and frequently recurrent. It may be due to granulosa cell tumor [4] or may be one symptom of the McCune-Albright Syndrome (MAS). MAS is a sporadic disease, characterized by the triad of polyostotic fibrous dysplasia, café-au-lait skin pigmentation, and endocrine dysfunction, with individuals exhibiting peripheral PP. It is due to post-zygotic-activating recurrent mutations in the guanine-nucleotide-binding protein (G protein) α-subunit (Gsα) [5]. The phenotype is variable and some patients carrying the mutation show only PP [5]–[9].

A number of other gene mutations could be responsible for peripheral PP. Women carrying homozygous inactivating *LHCGR* mutations have hypergonadotropic hypogonadism with primary amenorrhea or oligoamenorrhea, cystic ovaries, and infertility [10]. Mutations in the *FSHR* gene are associated with ovarian hyperstimulation syndrome that includes the presence of multiple serous and hemorrhagic follicular cysts [11]. 46,XX girls with mutations in the gene encoding Steroidogenic Acute Regulatory (StAR) protein spontaneously undergo puberty but ultimately develop ovarian cysts by an unknown mechanism [12]. A number of mouse knockouts also exhibit ovarian anomalies that may a genetic model for peripheral PP. The orphan nuclear receptor *NR5A1* (also known as steroidogenic factor 1) plays essential roles at multiple levels of the reproductive axis and controls the gonadal expression of multiple genes that are essential for reproduction [13]. Mice carrying a granulosa-specific *Nr5a1* knockout are sterile with ovaries that contain cysts [14]. Genes containing an evolutionary conserved DM domain are involved in various aspects of sexual development [15]. One member of the family, *DMRT4* is widely expressed during embryonic and postnatal development [15]. Mice lacking *Dmrt4* develop essentially normally, undergo full sexual differentiation in both sexes, and are fertile but females develop ovarian cysts [16]. Mice lacking *Nobox*, an oocyte-specific homeobox gene that is expressed in germ cell cysts and in primordial and growing oocytes, exhibit ovarian cysts at birth [17].

We analysed an exceptional series of 11 unrelated girls who presented with a prepubertal isolated ovarian cyst. In 4 cases there was a familial history of ovarian anomalies and/or infertility. We tested the hypothesis that mutations in the *GNAS1, LHCGR, FSHR, StAR*, *NR5A1, DMRT4* and *NOBOX* could contribute to the phenotype.

## Materials and Methods

### Ethics statement

All patients provided informed consent prior to participation in this study. The study was approved by local ethics committees. All clinical investigations were conducted according to the principals expressed in the Declaration of Helsinki.

### Patients

Written informed consent for the evaluation and molecular analyses was obtained from the parents. This study consisted of 11 girls referred to one of us (R. Brauner) with prepubertal isolated ovarian cyst. None of them had the other characteristic features of the neither MAS nor hypothalamic-pituitary lesion. Complete skeletal radiographic examination, and plasma concentrations of thyroid stimulating hormone, thyroxine, prolactin, β human chorionic gonadotropins and α-fetoprotein, measured at various intervals in each girl, were normal, as was the hypothalamic-pituitary area evaluated by magnetic resonance imaging.

The initial evaluation included determinations of height, growth rate [18], weight, pubertal stage [19], bone age [20], pelvic ultrasound examination, and measurement of plasma inhibin B (n = 4), anti-Müllerian hormone (AMH, n = 3), dehydroepiandrosterone sulfate, testosterone and delta 4 androstenedione concentrations. The hypothalamic-pituitary-ovarian axis was evaluated by measuring basal and GnRH (100 µg/m^2^)-stimulated LH and FSH peaks and the plasma estradiol concentrations. The biological evaluations were not complete in one girl with ovarian cyst diagnosed prenatally (Table 1). The values considered to be prepubertal were: uterus length of <35 mm [21], LH/FSH peaks ratio after GnRH test <0.66 [3], and plasma estradiol concentrations <15 pg/ml (55 pmol/l).

**Table 1:** Characteristics of 11 girls with prepubertal isolated ovarian cyst.

	First symptom		First evaluation										
							GnRH stimulation test			Pelvic ultrasonography			
							LH	FSH		Ovarian cyst	Uterus length		
Case	Age years	Type	Age years	Bone age years	Growth rate zs	Tanner stage	basal-peak, IU/l		Estradiol pmol/l	mm	mm	Evolution	Last evaluation age years
**1**	Prenatal	Ovarian cyst	1.3	ND	1	B2 P1	01–07	2.7–34.5	<37	Normal ovaries	25	L cystectomy	1.3
**2**	Prenatal	Ovarian cyst	1.7	ND	1	B1 P1	0.9–2.2	1.8–23.5	<37	Normal ovaries	27	Regression	1.7
**3**	Prenatal	Ovarian cyst	9.7	ND	1	B1 P1	ND	ND	ND	Normal R ovary	20	L ovariectomy	9.7
**4**	0.1	B2 M	2.1	2.8	0	B3 P1	0.5–4.3	1.6–14	ND	Normal ovaries*	ND	Recurrent M	23.5
**5**	0.1	B2 M	0.8	0.9	1	B2 P1	1–7.2	7–28	<37	Ovaries not seen	30	Recurrent M	21
**6**	1.5	M	2.2	3.5	2	B2 P1	<0.4–0.6	<0.4–0.8	9	L 17	36	Recurrent M	2.2
**7**	3.3	B2 M	3.3	ND	0	B3 P1	<0.1–<0.1	<0.1–1.1	66	L 50	57	CA treatment	6.4
**8**	5.6	B2 P2	5.7	4.5	1	B3 P2	<0.4–<0.4	<0.4–<0.4	918	L 60	53	CA treatment	13.5
**9**	5.8	B2	6.6	7.8	1.5	B3 P1	<0.2–0.74	<0.2–0.5	<37	Polycystic ovaries	34	Ovariopexy	9.5
**10**	6.6	B2	6.7	6.5	0	B3 P1	<0.4–<0.4	<0.4–<0.4	576	L 41	58	CA treatment	8.4
**11**	9.2	M	9.2	ND	1	B1 P1	<0.2–1.2	0.53–5	<37	Normal ovaries**	25	Recurrent M	11.3

*Ovarian cyst of 20 mm at 5 years; **blood effusion in the Douglas cul-de-sac.

B breast, P pubic hair, M vaginal bleeding, L: left; R right, ND, not determined, CA cyproterone acetate.

### Molecular analyses

Genomic DNA was extracted from peripheral blood leucocytes and ovarian cyst tissue (case 9) by standard methods. The exons 8 and 9 of the *GNAS1* gene were directly sequenced following PCR amplification essentially as described elsewhere with minor modifications [5]. Sequencing the gene coding for *NR5A1, LHCGR* and *FSHR* was performed as described elsewhere [13], [22], [23]. The primers and PCR conditions for amplification of the *StAR*, *DMRT4* and *NOBOX* are provided in supplementary material (Table S1). The recurring mutations at codons 201 and 227 of the *GNAS1* gene were screened by denaturating gradient gel electrophoresis (DGGE) analysis using chemical clamps instead of guanine-cytosine tails [5]. Computer algorithms were used to predict the fragment melting behavior and to determine the appropriate denaturant concentration range. An aliquot (1/10) of the amplified product was clamped by ultraviolet irradiation (365 nm) and loaded on a 6% polyacrylamide gel with a linearly parallel gradient of 30–60% denaturant (exon 7) or 50–80% denaturant (exon 9) and electrophoresed at 160 V (80 mA) for 7 hours. The amplified PCR products were purified and sequenced using the Dye terminator cycle sequencing kit (Perkin Elmer/Applied Biosystems, USA) and an ABI 377 machine.

## Results

Three patients were seen for history of prenatal ovarian cyst, 6 for breast development and 2 for vaginal bleeding all before 8 years of age (except case 11 aged 9.2 years, Table 1). All, except case 5, had a large unilateral ovarian cyst (17–60 mm) or polycystic ovaries (case 9) or a concomitant blood effusion in the Douglas cul-de-sac (case 11). In five patients, the plasma concentrations of LH and FSH were low and did not increase after GnRH stimulation. In five other cases plasma concentrations and response were prepubertal. They were not evaluated in case 3.

### Familial history and genetic analysis

**Figure 1:**
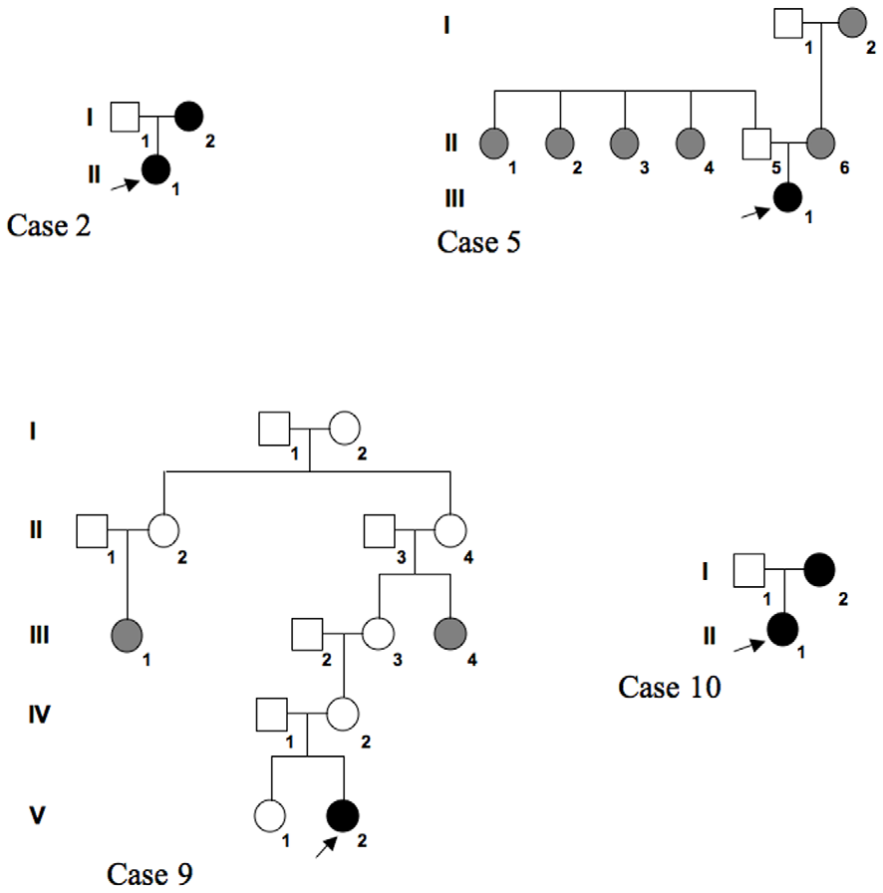
Pedigrees of 4 cases with familial history of ovarian anomalies. In case 2, the mother had an orange-sized ovarian cyst that was removed surgically in emergency at the age of 10 years. In case 5, four paternal aunts had vaginal bleeding and all were reported as infertile; in addition both the mother and maternal grandmother also reported menometrorragias. Hysterectomy was performed in the mother at 42 years because of a uterus fibroma diagnosed at 30 years. In case 9, the mother had an ovarian dermoid cyst at 25 years, leading to an ovariectomy; her mother's aunt was infertile, due to an undetermined ovarian trouble, after a menarche at the age of 10 years; her grand-mother's cousin underwent ovariectomy for an ovarian cyst. In case 10, the mother had ovarian cysts although there was no evidence of PP. Squares represent male family members, whilst circles represent female family members. The proband in each case is indicated by an arrow. Solid symbols indicate individuals with ovarian cysts. Shaded symbols indicate individuals with infertility and/or ovarian anomalies.

Four patients had a familial history of ovarian anomalies and/or sterility (Figure 1). These data suggest a strong genetic contribution to the phenotype in each case, but no mutations in the *GNAS1, LHCGR, FSHR, StAR*, *NR5A1, DMRT4* and *NOBOX* were found, including DNA extracted from the ovarian cyst tissue in case 9.

### Cases descriptions

Case 1 was referred for breast development at 1.3 years. A left ovarian cyst of 37 mm was diagnosed at ultrasonography performed at a gestational age of 33 weeks. At birth, it enlarged to 48 mm and was surgically removed at the age of 1 month. At 1.3 years, plasma basal LH and FSH concentrations and their response to GnRH test and estradiol were prepubertal. Ultrasonography showed normal ovaries. She has not been investigated since this time.

Case 2 was referred for a prenatal right ovarian cyst of 24 mm seen at ultrasonography performed at a gestational age of 33 weeks. It enlarged to 48 mm at 35 weeks and then diminished to 32 mm at birth. After birth, it progressively disappeared. Her mother also had a history of ovarian cyst (Figure 1). The patient was assessed at 1.7 years with no clinical evidence for PP. Plasma basal LH and FSH concentrations and their response to GnRH test were prepubertal. Plasma concentrations were prepubertal for estradiol and 22 pg/ml for inhibin B. Ultrasonography showed normal ovaries with small follicles. No medical problem has occurred in the last year since this assessment.

Case 3 was referred at 9.7 years for history of prenatal ovarian left cyst (37 mm) leading to ovariectomy for a necrotic ovary on the first day of life. The right ovary was initially polycystic and enlarged (30 mm) and then returned to normal. She has no pubertal development, and the clinical evaluation was normal. No GnRH test was performed and the current follow-up consists of an annual ultrasonography and clinical assessment.

Case 4 was referred for two episodes of breast development associated with vaginal bleeding at the first month of life and at 2 years. Plasma basal LH and FSH concentrations and their response to GnRH test were prepubertal. Plasma estradiol concentration was not measured, but the vaginal smear showed signs of estrogenisation. The plasma concentration of dehydroepiandrosterone sulfate was 110 ng/ml and progesterone was <0.05 ng/ml. Ultrasonography showed normal ovaries. Seven episodes of breast development occurred until the age of 5.8 years. Three of these were accompanied by vaginal bleeding and one was accompanied by abdominal pain, During these episodes, the ultrasonography showed a right ovarian cyst of 20 mm at 5 years and of 11 mm at 5.8 years. Since the vaginal bleeding was spontaneously regressive and the bone age was not advanced, she was not treated and no other bleeding episodes occurred. Menarche occurred at 13 years followed by regular menstruations. Adult height is 166 cm.

Case 5 had neonatal breast development and was referred for vaginal bleeding at 0.4 years. Plasma basal LH and FSH concentrations and their response to GnRH test and estradiol were prepubertal. Ultrasonography showed a uterine vacuity line, while the ovaries were not seen. Other plasma concentrations were dehydroepiandrosterone sulfate 130 ng/ml and progesterone <0.05 ng/ml. Five episodes of vaginal bleeding occurred until the age of 2.8 years. Gynecologic examination and complete haemostasis tests were normal. At 9.5 years, another episode of vaginal bleeding occurred. Gonadotropins response to the GnRH test was again prepubertal (peaks LH 3.6 and FSH 14.3 IU/l, with plasma concentrations of 37 pmol/l for estradiol, 6.5 pmol/l for AMH and <10 pg/ml for inhibin B). Ultrasonography showed round ovaries without a cyst. Bone age was 10 years. She was then lost for follow-up for 10 years. During this period she reported irregular menstrual cycles. Adult height is 173 cm. At 21 years, she had regular menstrual cycles due to estro-progestative contraception. Plasma basal concentrations measured after stopping this were LH 9.4 IU/l, FSH 9.9 IU/l, testosterone 0.24 ng/ml and delta-4-androstenedione 1.2 ng/ml. Ultrasonography showed normal ovaries. As four paternal aunts were infertile (Figure 1), her chromosome complement was analyzed and was 46,XX.

Case 6 was referred for breast development and vaginal bleeding at 2.2 years. Previous vaginal bleeding had occurred at the age of 1.5 years, but it did not lead to a medical evaluation. Plasma basal LH and FSH concentrations were low and they did not increase after GnRH stimulation test, while estradiol was prepubertal with inhibin B<10 pg/ml. Ultrasonography showed a left ovarian cyst and a normal right ovary. There was no evidence for a tumoral mass neither by pelvic magnetic resonance imaging nor by blood tests.

Case 7 was referred for breast development at 3.3 years, with areolas pigmentation, followed one month later by an episode of vaginal bleeding. She was one of two non-identical twin girls, born at 28 weeks of gestation, weighting 0.740 kg. She had prolonged ventilation. Plasma basal LH and FSH concentrations were low and did not increase after GnRH stimulation. Ultrasonography showed a left ovarian cyst, a normal right ovary and a pubertal uterus length. Because of the large dimension of the cyst, treatment by oral cyproterone acetate was initiated at the dose of 25 mg (1.8 mg/kg)/day. Estrogenization signs disappeared in 5 months. The ovarian cyst decreased, measuring 28 mm at 4.7 years and then disappeared. Treatment is being continued, at a decreased dose of 12.25 mg (0.8 mg/kg)/day since the age of 4.3 years. Ultrasonography did not show ovarian cyst at 6.1 years.

Case 8 was referred for breast and pubic hair development at 5.6 years. For more than one year, she had intermittent clear vaginal discharge. Plasma basal LH and FSH concentrations were low and did not increase after GnRH stimulation test, while estradiol was very high. Ultranography showed a left ovarian cyst, normal right ovary with small follicles, and a pubertal uterus length. Because of the large dimension of the cyst, treatment by oral cyproterone acetate was initiated at the dose of 75 mg (3 mg/kg)/day. The ovarian cyst disappeared after 7 months. Discontinuation of treatment was followed by resurgence of clear vaginal discharge less than 2 months later. Treatment was resumed at the dose of 25 mg/day. At 7.5 years, a 19x10mm left ovarian cyst was seen at ultrasonography, with a uterus length of 40mm. There was no clinical sign of estrogenisation. The treatment dosage was increased to 50 mg (0.9 mg/kg)/day until 10.5 years. Plasma estradiol concentration was <37 pmol/l at 9.8 years. Menarche occurred at 11.5 years followed by regular menstruations.

Case 9 was referred for breast development at 5.8 years, with a familial history of ovarian anomalies (Figure 1). Plasma basal concentrations of LH and FSH were low and and did not increase after GnRH stimulation test, while the estradiol level was <37 pmol/l. Ultrasonography showed polycystic ovaries. During the initial follow-up, ultrasonography showed no modification. She was not treated because breast development and bone age did not progress. Plasma concentrations were testosterone <0.05 ng/ml and delta-4-androstenedione 0.74 ng/ml at 8.8 years. At 9.3 years, her Tanner stage was B4 P1, and ovaries size was increased, with a larger diameter of 60 and 70 mm and many cysts, measuring 8 to 12 mm. The plasma concentrations were 381 pmol/l for AMH and 94 pg/ml for inhibin B. At 9.4 years, ovariopexy and multiple ovarian biopsies were performed: macroscopically, both ovary diameters were more than 100 mm, and there were multiple cortical cysts, with no sign of malignancy. Plasma estradiol concentration in the cyst fluid was 1945 pmol/l.

Case 10 was referred for breast development with areolas pigmentation and a clear vaginal discharge at 6.6 years. There is a familial history of ovarian anomalies (Figure 1). Plasma basal LH and FSH concentrations were low and did not increase after GnRH stimulation test, whilst plasma estradiol concentration was high. Ultrasonography showed a left ovarian cyst, a normal right ovary with small follicles and a pubertal uterus length. Because of the large dimension of the cyst, treatment by oral cyproterone acetate was initiated at the dose of 50 mg (1.8 mg/kg)/day. Three months later, breast development decreased, the ovarian cyst disappeared, the plasma concentration of estradiol decreased to <37 pmol/l, while AMH was 25 pmol/l and inhibin B <10 pg/ml. The dose was when decreased to 12.25 mg (0.5 mg/kg)/day. At 8.4 years, she remained under the same treatment, her bone age was 8.9 years, and the ultrasonography was normal.

Case 11 was referred for vaginal bleeding at 9.2 years. There was no breast or pubic hair development. Plasma basal LH and FSH concentrations and their response to GnRH test and plasma estradiol concentrations were prepubertal. Ultrasonography showed normal ovaries. The following two years were characterized by periodic vaginal bleeding, once a month and then twice a month in the last year. Iterative pelvic ultrasonographies did not reveal any ovarian cyst but indirect evidence for it was seen by a blood effusion in the Douglas cul-de-sac.

## Discussion

Here, we have described the clinical-biological presentation and genetic analyses of 11 rare cases of precocious puberty with ovarian cysts. “Idiopathic” ovarian PP shows markedly different features from central PP. It raises the questions of differential diagnosis from ovarian tumor, the risk of developing ovarian torsion and influencing bone age progression induced by the estrogen secretion that can lead to a short adult height.

### Clinical and biological presentation

The presentation of the 8 postnatally diagnosed cases differs from the typical presentation of central PP. Signs of estrogenisation were intense, developed quickly and they were associated with vaginal bleeding in 5 of the cases. In 5 of the 8 cases the plasma basal LH and FSH concentrations were low and did not increase after GnRH stimulation. Their evolution was also unusual with spontaneous regression in 4 cases with recurrent episodes. In our experience, in patients with central PP, vaginal bleeding occurs associated with hypothalamic hamartoma or suprasellar arachnoid cysts but it does not occur in idiopathic forms [2], [24]. These central PP cases are associated with pubertal gonadotropin response to the GnRH test [25]. In the patients of the present study, a hypothalamic-pituitary lesion was excluded by magnetic resonance imaging and none had pubertal response to GnRH stimulation test. The cysts in the patients here were all greater than 17 mm diameter. An isolated ovarian cyst of more than 8–10 mm diameter suggests a peripheral origin of the PP, while in central puberty both ovaries are characteristically enlarged and multifollicular [26], [27]. This would explain the rapid estrogenisation signs followed by regression. The short time of the secretion of estrogens, even if repeated, probably explains the absence of a significant bone age progression leading to a normal adult height reached by the two older patients.

### Diagnosis and prognosis

Peripheral PP may be the first symptom of an ovarian tumor, including granulosa cell tumor, which is present in 1% of all peripheral PP [28]. In a previous study of prepubertal girls with ovarian granulosa cell tumor [4], 17/29 presented with PP. However, two girls were misdiagnosed with ovarian cyst, since this tumor is frequently heterogeneous and can have a cystic component [29]. This is important, because a delayed diagnosis is known to worsen the overall prognosis [29]. In our series, a tumor was suspected in case 9 but surgical biopsies eliminated this diagnosis. In the other patients, the disappearance of the ovarian cysts suggests tumors are absent. Plasma AMH and inhibin B concentrations were measured in the 4 more recent cases because granulosa cell tumors may secrete these hormones [30] and AMH is increased in prepubertal daughters of women with polycystic ovary syndrome [31]. In 4 cases, the symptoms regressed spontaneously. In three cases, the treatment with cyproterone acetate was associated with regression of clinical-biological-radiological abnormalities. The risks of ovarian torsion due to the volume of the ovarian cyst and of recurrence of estrogenic signs required a careful follow-up.

### Molecular analyses

The genetic basis of ovarian precocious puberty is unknown. The presentation of the 11 cases may form part of the clinical spectrum of the MAS and, therefore they may carry activating mutations in the *GNAS1* gene. A mutation screen in all cases failed to detect a mutation. However, in 10/11 cases lymphocyte DNA was analyzed and a post-zygotic somatic mutation confined to the ovaries cannot be excluded. A previous study found a *GNAS1* mutation in the cystic fluid of a girl with isolated peripheral PP who had no other clinical presentation of MAS during a 40 months follow up [8]. However, bone anomalies were not excluded and the patient did not respond to cyproterone acetate treatment, which suggests a different clinical presentation to the cases reported here. In a study of 39 girls presenting isolated peripheral PP [5], *GNAS1* mutations were observed in 13 cases (33%). Five of these patients did not show either skin or bone lesions during their follow up, or were lost to follow up. In a separate study somatic MAS features also developed in 3 patients two years (15–40 months) after vaginal bleeding [32]. Here, *GNAS1* mutations were not found in ovarian cystic tissue of case 9. In the remaining cases of our study the mean follow up period is 10.9 years, yet none of the patients developed characteristic MAS anomalies. A familial history of ovarian anomalies was reported in four of the cases reported here also arguing against the involvement of the *GNAS1* gene.

We screened a series of candidate genes for mutations that could be associated with the development of ovarian cysts. Mutations of the *LHCGR* and *FSHR* genes may result in the development of ovarian cysts or hyperfunction [10], [11], however in this study we did not identify mutations in either of these genes. Based on the mouse knockout phenotypes, mutations involving the *NOBOX, DMRT4, NR5A1* or *StAR* genes could be associated with cyst formation, however we did not detect mutations in the coding sequences of these genes in any of the cases described here. Although we did not detect mutations in these genes it is probable that a genetic factor is contributing to the phenotype since in several cases there was a family history of ovarian anomalies.

## Supporting Information

Table S1 List of primer sequences and PCR cycling conditions used for NOBOX, DMRT4 and STAR genes in the study. In each amplication, 37 cycles were performed using 10ng of genomic DNA.
(0.07 MB DOC)
