# Supplementary material for: Clinical, Biological and Genetic Analysis of Prepubertal Isolated Ovarian Cyst in 11 Girls
Source: PLoS One. 2010 Jun 25;5(6):e11282. doi: 10.1371/journal.pone.0011282 (PMC2892512; doi:10.1371/journal.pone.0011282)
Supplement: Table S1 — List of primer sequences and PCR cycling conditions used for NOBOX, DMRT4 and STAR genes in the study. In each amplication, 37 cycles were performed using 10ng of genomic DNA. (0.07 MB DOC) [file pone.0011282.s001.doc]

| Primers | Sequence 5’ → 3’ | Cycling conditions |
| --- | --- | --- |
| NOBOX F1 | TCCCAGCTTCTAACCACAC | 95°C→30 sec  56°C→30sec  72°C→60sec |
| NOBOX R1 | CAATTTTGCTGTTGCTTCCA |
| NOBOX F2 | GATCTCCCCCAAGAAAGTCC | 95°C→30 sec  64°C→30sec  72°C→60sec |
| NOBOX R2 | GGCTAGGCTGGGCTAGAACT |
| NOBOX F3 | GCCTGGAGACAATCGAATCCT | 95°C→30 sec  64°C→30sec  72°C→60sec |
| NOBOX R3 | ACGGCGTTAGCTCATGGTAT |
| NOBOX F4 | GAAGCAGCACTTCCTCTTGG | 95°C→30 sec  64°C→30sec  72°C→60sec |
| NOBOX R4 | CAAACTAACCCCATCCCTCA |
| NOBOX F5 | GCCCCATCTGTTGGAGATAC | 95°C→30 sec  64°C→30sec  72°C→60sec |
| NOBOX R5 | AGCCTTCCAATGGTCTCCTT |
| NOBOX F6 | AGGAGGCAGAGTTTGGGAAT | 95°C→30 sec  64°C→30sec  72°C→60sec |
| NOBOX R6 | TCCCCTTATCCCTTTCATCC |
| DMRT4 F1 | CTCCCAGCAGGGTTAGCTG | 95°C→30 sec  62°C→30sec  72°C→60sec |
| DMRT4 R1 | CGATCAGGGTGCACTTGG |
| DMRT4 F2 | CGTAACCATGGTGTGGTGTC | 95°C→30 sec  62°C→30sec  72°C→60sec |
| DMRT4 R2 | AACTATTCCTGCCCGCCTAT |
| DMRT4 F3 | GGAGTGAATTAATTATATTATCCAGCA | 95°C→30 sec  62°C→30sec  72°C→60sec |
| DMRT4 R3 | CAGTTCTTCTGAGTTTGCTAGGTTC |
| DMRT4 F4 | AAGGCATTCTACGGTTCTGC | 95°C→30 sec  62°C→30sec  72°C→60sec |
| DMRT4 R4 | CCCAGCATTTTAAGACCTCA |
| STAR F1 | GGGGACATTTAAGACGCAGA | 95°C→30 sec  61°C→30sec  72°C→60sec |
| STAR R1 | CCCAGGTTCACCCAGTAAGA |
| STAR F2 | CACTTCCCTCTCCAAACCAA | 95°C→30 sec  61°C→30sec  72°C→30sec |
| STAR R2 | GGGACGTCCTCTCAAAATGA |
| STAR F3 | AGTTCCTCAAGGCCAGATCC | 95°C→30 sec  61°C→30sec  72°C→30sec |
| STAR R3 | GAGGAACCACAGGCTTCTCC |
| STAR F4 | GCTGGGATTATAGGCGTGAA | 95°C→30 sec  66°C→30sec  72°C→30sec |
| STAR R4 | TGCAGGCCTGTGTTAGAAGA |
| STAR F5 | CTGTGCCAACTGCCAAATAA | 95°C→30 sec  61°C→30sec  72°C→30sec |
| STAR R5 | CTCACTACCACCTGCCTTCC |
| STAR F6 | ACAGAGCTCGAAGACCAAGC | 95°C→30 sec  61°C→30sec  72°C→30sec |
| STAR R6 | CATCCCACTGTCACCAGATG |
